# Prevalence, Distribution, and Molecular Record of Four Hard Ticks from Livestock in the United Arab Emirates

**DOI:** 10.3390/insects12111016

**Published:** 2021-11-11

**Authors:** Nighat Perveen, Sabir Bin Muzaffar, Mohammad Ali Al-Deeb

**Affiliations:** Department of Biology, United Arab Emirates University, Al-Ain 15551, United Arab Emirates; 201790740@uaeu.ac.ae (N.P.); s_muzaffar@uaeu.ac.ae (S.B.M.)

**Keywords:** 16S rRNA, cytochrome oxidase subunit 1, *Amblyomma lepidum*, *Hyalomma anatolicum*, *Hyalomma dromedarii*, *Rhipicephalus sanguineus*, prevalence, livestock

## Abstract

**Simple Summary:**

Ticks, as blood feeders and vectors of many diseases, can negatively impact livestock and human health, with potential economic impacts on the livestock industry. In this study, we documented the occurrence of four tick species (*Hyalomma dromedarii*, *Hyalomma anatolicum*, *Rhipicephalus sanguineus*, and *Amblyomma lepidum*) on camels, cows, sheep, and goats from three areas in the United Arab Emirates (UAE). Our findings indicated that *H. dromedarii* was the most prevalent tick species on camels. The other tick species were present at varying levels on hosts. Some of the tick species collected in this study are potential carriers of tick-borne diseases that are serious and sometimes fatal to humans and animals. Thus, there is a need for more research on ticks and tick-borne diseases in the UAE.

**Abstract:**

Ticks are important arthropod vectors that serve as reservoirs of pathogens. Rapid urbanization and changes in animal breeding practices could be causing a rise in tick burden on animals. Studies on tick distribution on livestock and tick molecular diversity from the United Arab Emirates (UAE) are limited. The aim of this study was to (i) provide molecular and morphological identification of tick species, (ii) compare tick infestation between different hosts, (iii) compare tick infestation in relation to the sex of the host, and (iv) assess the prevalence of tick species on hosts. A total of 5950 ticks were collected from camels (4803 ticks), cows (651 ticks), goats (219 ticks), and sheep (277 ticks). Ticks were identified based on morphological characters at the species level using taxonomic keys. In addition, Polymerase Chain Reaction (PCR) amplification of the cytochrome oxidase subunit 1 (cox1) and 16S rRNA mitochondrial genes was used to identify ticks. Four species were confirmed based on molecular and morphological characterization, namely, *Hyalomma dromedarii*, *Hyalomma anatolicum*, *Rhipicephalus sanguineus*, and *Amblyomma lepidum*. *Hyalomma dromedarii* (94.3%) was the most abundant species, followed by *H. anatolicum* (32.8%). Camels were heavily infested (94%) with ticks as compared to cows (38%), sheep (37%), and goats (14%). Widespread occurrence of these four tick species in the UAE poses a risk of spreading tick-borne pathogens wherever the conditions of infection prevail.

## 1. Introduction

Hard ticks (Acari: Ixodoidea) are significant ectoparasites of livestock and transmit many pathogens of concern to public health and veterinarians in the Middle East and North Africa (MENA) region [1,2]. They are second to mosquitoes in significance as disease vectors [3] and are responsible for substantial economic, social, and conservation losses due to their harmful effects on human and animal health [4]. Ticks and tick-borne diseases (TBDs), through their impact on livestock and human health, result in losses of US $13.9–18.7 billion annually [5]. Livestock are imported annually in large numbers from countries such as Sudan, Somalia, Turkey, Argentina, Pakistan, Australia, Iran, India, and Uruguay to Saudi Arabia and the UAE. Camel, sheep, goat, and cattle production plays a fundamental role in the agricultural sector and contributes substantially to the food security of the country [6]. Prior to 1994, tick records from the UAE were scarce. Ticks collected during the Crimean-Congo hemorrhagic fever (CCHF) outbreak in 1994–1995 resulted in reporting 14 tick species from the UAE. Most CCHF virus-infected animals in this outbreak were imported from Somalia, with fewer numbers of infected animals possibly arriving from Iran [7]. Thus, monitoring tick infestations of imported animals is an important priority.

*Hyalomma* (Acari: Ixodidae) ticks are widespread in the MENA region and transmit a variety of viral, bacterial, and parasitic diseases among animals and humans [2,8]. For example, *Hyalomma* species have been associated with tick paralysis in humans [9]. *Hyalomma dromedarii* is of particular importance in the region, being the most prevalent species in the MENA region [10,11,12,13,14]. The adult tick of this species preferentially feeds on camels as the primary host. It infests other domestic animals, such as cattle, sheep, goats, and equines, albeit at lower prevalence [10]. It completes its lifecycle on two or three hosts [11] and can be found feeding on camels throughout the year in the UAE [12]. The adults of another widespread species, *Hyalomma anatolicum*, feed on cattle, camels, horses, sheep, and goats. Their immature stages may also feed on small mammals and even humans associated with livestock husbandry [11]. It behaves as a two-host or three-host tick depending on the presence of hosts, and may be found throughout the year [11]. However, *Rhipicephalus sanguineus* is a kennel tick, though it can be found on cattle [11] and occasionally feeds on humans [15]. *Amblyomma lepidum* is another important hard tick species affecting livestock, which preferentially feeds on cattle or camels [11]. It is widely distributed in Sudan, Ethiopia, Somalia, Uganda, Kenya, and Tanzania.

Ticks can be identified at the species level based on morphological characters and molecular markers. Accurate identification of ticks is important to better understand tick species ecology, life cycle, and tick-borne disease epidemiology. Different molecular markers have been used to determine the phylogeny of ticks [16] including 16S rDNA, 12S rDNA, cox1, 18S rDNA, 28S rDNA, ITS1 rDNA, and ITS2 rDNA [16,17]. The precise identification of tick species is essential for implementing adequate control measures against the spread of tick-borne diseases. Morphological identification relies mostly on characterizing size, color, and structure of tick mouth parts (hypostome, palps, etc.), scutum, cervical and lateral grooves, festoons, ventral plates, and spiracles [14]. However, the taxonomical identification of ticks based on morphological features can pose significant challenges. Firstly, the structure of the genital opening is not clear enough to differentiate the tick species in gravid females (that are distended with eggs). Secondly, tick specimens may have diagnostic taxonomic parts, such as scuta and mouthparts, either damaged or lost. Thirdly, the immature stages may require high-power microscopes or even scanning electron microscopes for proper identification at the species level [18,19]. Although many keys are available as aids, morphological identification of tick species remains difficult and expert knowledge is required. In addition, incorrect identification at the level of species and subspecies is common, particularly for complex members, such as *H. anatolicum* and *H. excavatum*. The high similarity between these two species is the cause of much confusion in the literature [20]. Therefore, molecular characterization of ticks using genetic markers has gained superiority in recent years [21,22,23]. The 16S rRNA and cox1 are useful markers for tick taxonomy at the species level [22,24]. So far, very few studies in the UAE have provided molecular records of ticks [25,26]. For example, *H. dromedarii* was identified based on morphology, and its identification was confirmed based on the use of the cox1 gene [25]. Furthermore, the genetic diversity of several populations of *H. dromedarii* in the UAE was determined using the cox1 gene and Randomly Amplified Polymorphic DNA Polymerase Chain Reaction (RAPD-PCR) [26]. Thus, molecular tools provide an opportunity to correctly identify tick fauna in the UAE.

The aim of this study was to (i) provide molecular and morphological identification of tick species, (ii) compare tick infestation between different hosts, (iii) compare tick infestation in relation to the sex of the host, and (iv) assess the prevalence of tick species on hosts.

## 2. Materials and Methods

### 2.1. Ethical Approval

This study was carried out in accordance with recommendations of the Animal Research Ethics Committee (A-REC) of the UAE University (ethical approval ERA_2019_5953). In addition, the experimental protocol was approved by the UAE University Research Office.

### 2.2. Study Site

**Figure 1:**
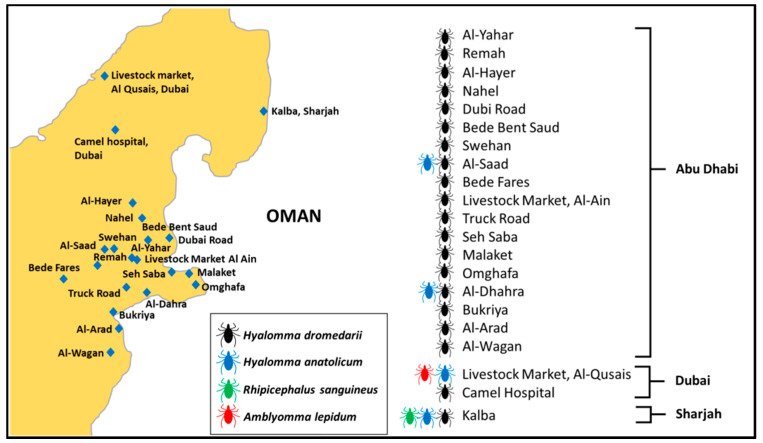
Tick collection sites and distribution of tick species on livestock in the study area, in 2019, 2020, and 2021, in the United Arab Emirates.

This study was conducted mainly in two types of areas: open farms and closed animal markets. The first type included open farms in desert regions within the UAE where livestock holding areas are accessed by large and small mammals, reptiles, and birds. Most of these areas also lie at the border of Oman. In addition, sampling was done at areas in which animals were reared on farms and housed in a homestead, locally called an izba. In general, the open areas have a typical desert ecosystem climate, which is characterized by high amplitudes of seasonal temperatures with mean monthly temperatures varying between 17.1 °C in winter and 38.1 °C in summer [27]. The area is the home to a camel farming community where keeping livestock is a way of life. The second type included two livestock markets in Dubai and Al Ain (Figure 1). The details about the animal hosts sampled, the distribution of samples among the sites, tick species recorded at each site, and the years when each site was sampled are given in Supplementary Table S1.

### 2.3. Tick Sampling

We used a cross-sectional study design for this work. Animals were sampled from three emirates in the UAE, namely, Abu Dhabi, Dubai, and Sharjah (Figure 1). At desert farms, tick collection from animals was undertaken either early in the morning or in the evening. A total of 587 domestic animals were examined, including 300 camels, 119 cows, 97 sheep, and 71 goats. A total of 5950 ticks were collected from the bodies of 587 animals with 4803 ticks collected from camels, 651 ticks from cows, 219 ticks from goats, and 277 ticks from sheep. Most ticks were collected from perianal and vulvar regions, the inner surface of thighs, udders, and inguinal regions. All ticks were removed from the entire body of each examined animal manually using forceps and placed in 50 mL plastic vials. The vials containing ticks were retained inside an icebox and were taken to the Animal Ecology and Entomology Laboratory at UAE University, where they were frozen at −80 °C until further processing. All vials were labeled, and the ticks were counted. Labeling for all samples included location, host, date of sampling, number of ticks, and the gender of the host (male or female).

### 2.4. Morphological Identification of Ticks

In the laboratory, the sampled ticks were rinsed with 70% ethanol and deionized water for five minutes to remove environmental particulate contamination [28] and then air dried. Ticks were identified based on morphological characteristics under a dissecting Nikon SMZ1500 Stereoscopic zoom microscope (Nikon, Tokyo, Japan) at the species level using taxonomic keys [11,29,30,31,32] and sorted according to sex and stage of development.

### 2.5. Molecular Characterization of Ticks

#### 2.5.1. DNA Extraction

After identification, DNA was extracted from 44 individual ticks (11 ticks from each host) to confirm their identification and provide a molecular record in the GenBank. Briefly, legs were removed from the ticks with a sterile scalpel blade and homogenized in a 1.5 mL tube using liquid nitrogen. DNA extraction was performed with a DNA Mini Kit (Qiagen, Hilden, Germany) following the manufacturer’s protocol. The DNA concentration of each sample was estimated using a NanoDrop 2000 UV spectrophotometer (Thermo Fisher Scientific, Waltham, MA, USA). Extracted DNA samples were stored in freezer at −20 °C.

#### 2.5.2. Polymerase Chain Reaction Amplification

DNA was subjected to polymerase chain reaction to amplify regions of the cox1 and the 16S rRNA genes. Gene fragments were separately amplified from all 44 samples using specific oligonucleotide primer pairs (Table 1) to amplify 710 bp of the cox1 gene and 460 bp of the 16S rRNA gene based on published protocols [33,34]. The PCR amplifications were performed in a Swift MaxPro thermo-cycler (ESCO, Singapore) and each PCR was carried out in 25 μL reaction volume containing 12.5 μL of Taq PCR master mix (Qiagen, Hilden, Germany), 1.0 μL (10 pM) of each primer, 3.0 μL of tick genomic DNA, and 7.5 μL of nuclease free water. Thermo-cycle conditions are given in Table 1. In each PCR a negative control (no-template DNA) was used to detect contamination and a positive control was used to confirm that primers were properly annealing to the target region on the template DNA.

#### 2.5.3. Agarose Gel Analysis, Amplicon Purification and Sequencing

Amplicons were visualized in 1.5% agarose gel (Promega, Madison, WI, USA). Further, they were purified using QIAquick PCR Purification Kit (Qiagen, Hilden, Germany) according to manufacturer’s protocol and DNA concentration was measured using a spectrophotometer, NanoDrop 2000 UV spectrophotometer (Thermo Fisher Scientific, Waltham, MA, USA). DNA fragments were sequenced by Sanger Sequencing at the Sequencing Unit, Biology Department, UAE University.

#### 2.5.4. DNA Sequence Analysis

Sequences of the cox1 and 16S rRNA genes from the present study were compared with the available data in the GenBank using the Basic Local Alignment Search Tool (BLAST) on the National Center for Biotechnology Information (NCBI) website (https://blast.ncbi.nlm.nih.gov/Blast.cgi (accessed on 28 July 2021)). DNA sequence analysis was performed based on DNA sequence similarity using BLAST, for all four species, *H. dromedarii*, *H. anatolicum, R. sanguineus*, and *A. lepidum.* Representative sequences from this study, were deposited in the GenBank database.

#### 2.5.5. Statistical Analysis

The number of ticks was recorded on camels, cows, sheep, and goats. The prevalence (proportion of hosts infested with ticks), mean intensity (number of ticks per infested host), and mean abundance (number of ticks per host) were calculated for all hosts [35]. Mean intensities and mean abundance values were compared between hosts using bootstrap *t*-tests, and the *p*-values were generated using 2000 replications. The prevalence of ticks was compared between hosts and within the same host on the basis of sex using Fisher’s exact test and 95% confidence levels using the Clopper–Pearson method. All comparisons were made using the Quantitative Parasitology Software Version 3.0 [35].

## 3. Results

### 3.1. Tick Identification

Four species of ticks in three genera, namely, *H. dromedarii*, *H. anatolicum*, *A. lepidum* and *R. sanguineus* were identified based on morphology. In addition, the species designation was confirmed using DNA sequencing.

**Figure 2:**
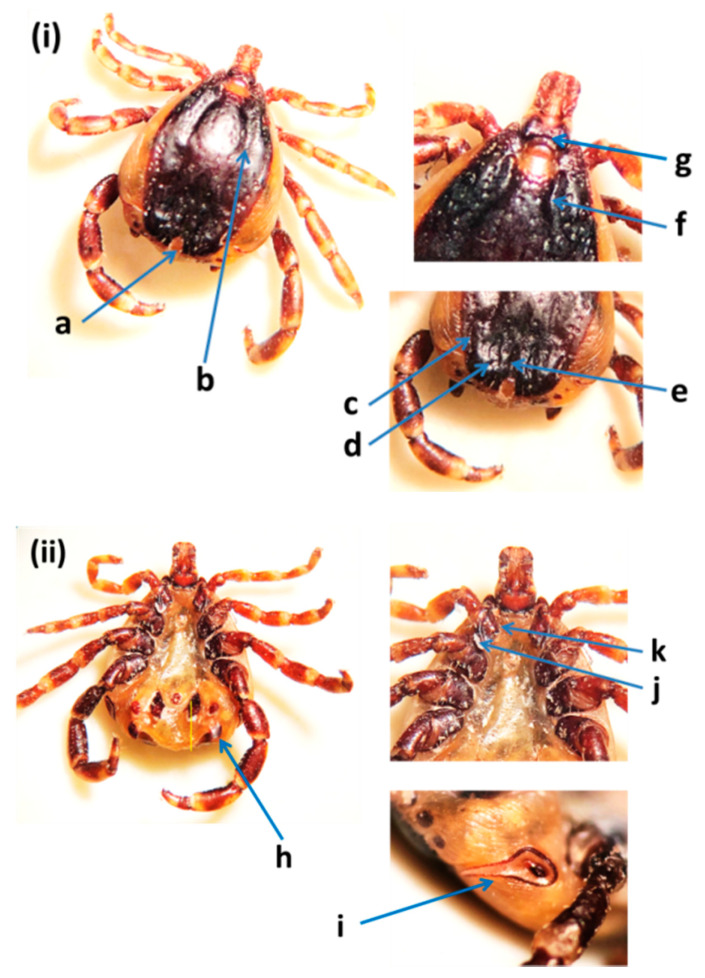
*Hyalomma dromedarii* male from the United Arab Emirates: (**i**) dorsal view: (a) central festoon, (b) cervical and lateral grooves, (c) marginal grooves, (d) paramedian grooves, (e) posteromedian groove, (f) cervical grooves, (g) basis capituli; (**ii**) ventral view: (h) sub-anal plates, (i) spiracular plates, (j) posterolateral spurs, (k) posteromedian spur [11,32].

The identification of *H. dromedarii* was confirmed based on the following diagnostic characteristics: the sub-anal plates were aligned outside the adanal plates in male ticks (Figure 2); the central festoon was pale colored; cervical and lateral grooves reached up to 2/3 the length of the conscutum; the marginal grooves were short and furrow-like; the paramedian grooves were well defined and large; the posteromedian groove reached the parma; the cervical grooves were very deep; the dorsal posterior margin of the basis capituli was deeply concave; the dorsal prolongation of spiracular plates were long and narrow; and the posterolateral spurs were longer than the posteromedian spur and they were tapered at the apices. *Hyalomma dromedarii* was identified in all three emirates from camels using cox1 and 16S rRNA genes. The species was confirmed based on DNA sequence similarity with GenBank records (Supplementary Table S2). A representative sequence of *H. dromedarii* from camels in this study was submitted in the GenBank (MZ976772) (Table 2). The UAE specimen showed 99.50% similarity to the sequences of *H. dromedarii* detected from camels in Tunisia (MN960589.1) and Egypt (MG757400.1), with sequence coverage of 95% (Supplementary Table S2).

**Figure 3:**
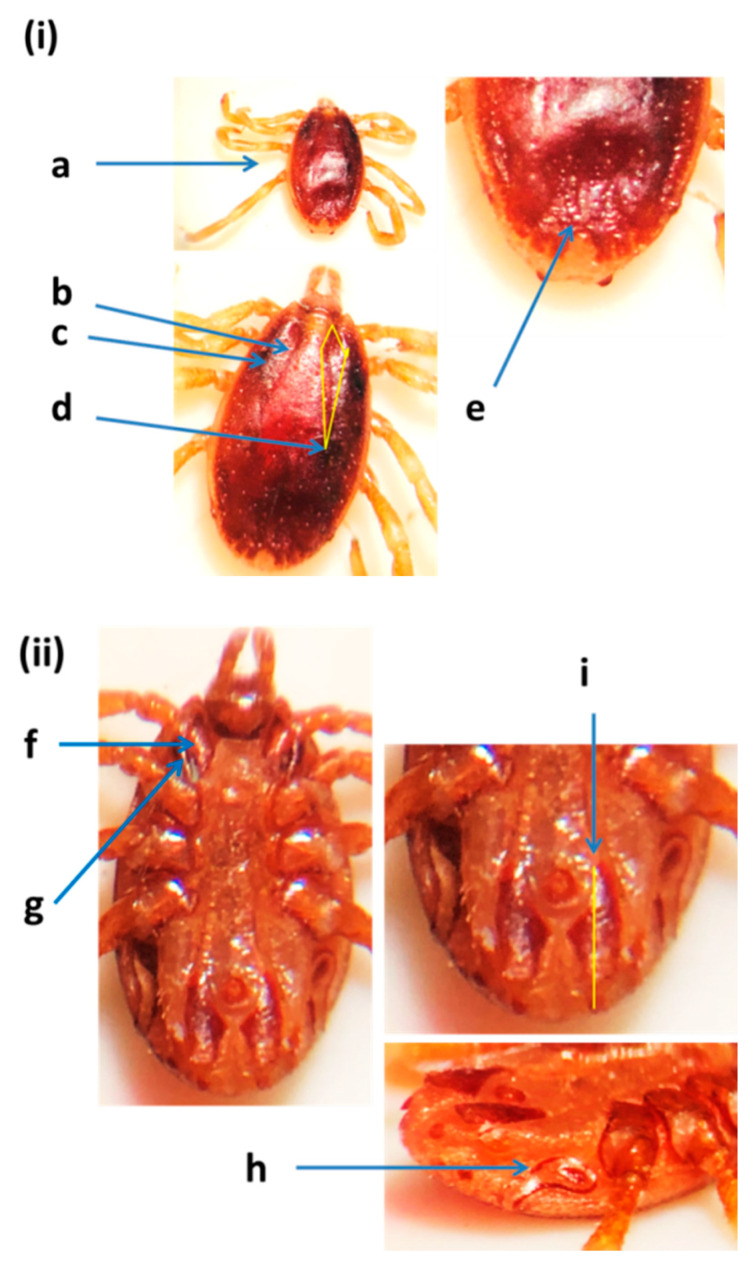
*Hyalomma anatolicum* male from the United Arab Emirates: (**i**) dorsal view: (a) reddish-brown and oval shape, (b,c) cervical grooves and lateral grooves, (d) cervical and lateral grooves length, (e) posteromedian groove; (**ii**) ventral view: (f) coxae I spurs close together, medial spur in the form of a triangle, (g) coxae I lateral spur (narrow), (h) spiracle plate, (i) subanal shields situated on the axis of the adanals [11,31].

*Hyalomma anatolicum* ticks were small in size (about 2.69 mm in length), oval in shape, and reddish brown in color (Figure 3). Cervical grooves and lateral grooves were shallow and reached 1/2 the length of the conscutum and the posteromedian groove did not reach the parma. The spurs of Coxa I were close together and the medial spur was wider than the lateral and was trianglular. The lateral spur of Coxae I was narrow and curved. The sub-anal shields were situated on the axis of the adanals. *Hyalomma anatolicum* was identified from camels, cows, sheep, and goats in the three emirates using 16S rRNA and cox1 genes. However, it was not detected in tick samples collected from camels in Dubai and Sharjah. DNA fragments were identified based on sequence similarity with the records of both 16S rRNA and cox1 genes from the GenBank (Supplementary Table S3). Representative sequences of *H. anatolicum* from cows, sheep, and goats (MZ976771, MZ976770, and MZ976780) for the 16S rRNA gene and from cows (OK017169) for the cox1 gene were deposited in the GenBank (Table 2). This sequence was 99.70% identical to the *H. anatolicum* detected from goats in Pakistan (MT800311.1), and 99.39% similar to *H. anatolicum* reported from China (MH459380.1) with sequence coverage of 96% and 97%, respectively (Supplementary Table S3).

**Figure 4:**
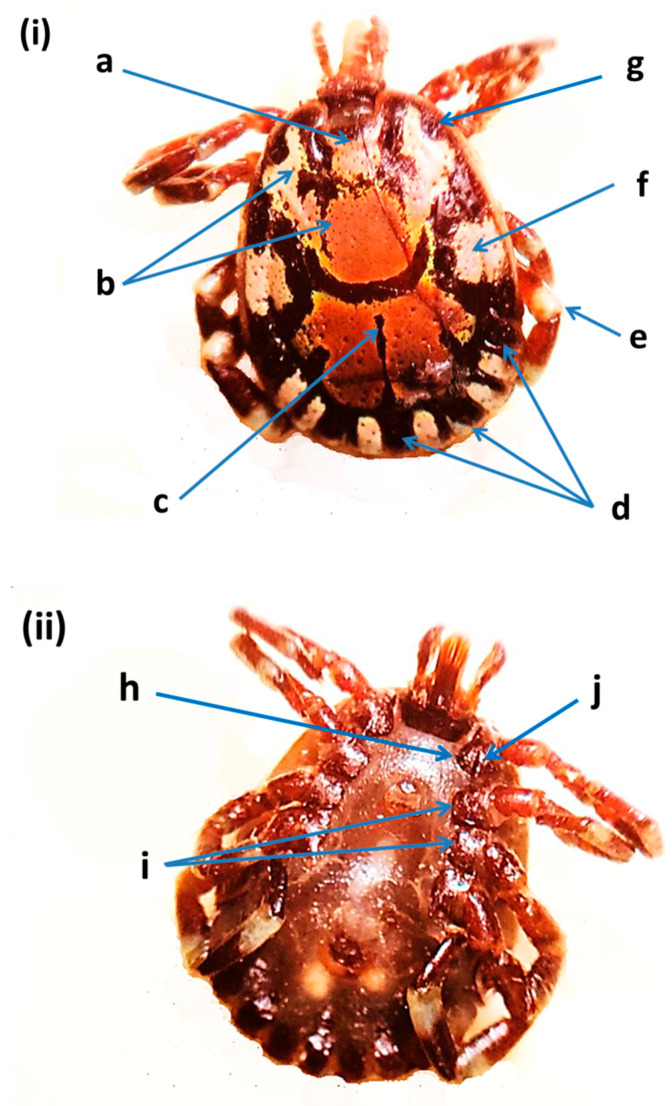
*Amblyomma lepidum* male from the United Arab Emirates: (**i**) dorsal view: (a) primary punctuation, (b) enamel color, (c) posteromedian strip, (d) festoon enameling, (e) leg with pale ring, (f) lateral median areas of enamel orientation, (g) eyes (distinctly convex); (**ii**) ventral view: (h) coxae I internal spur length, (i) coxae II and III, (j) coxae I external spur length [11,30].

*Amblyomma lepidum* found in the UAE (Figure 4) was a large and ornamented tick with long mouthparts. Diagnostic features are given in Figure 1. Primary punctuation size was small and distribution was localized (between the eyes). Enamel color was pink to orange. The posteromedian strip was narrow. Enameling of the festoons was partial (no enamel on the central and two outermost festoons). Leg coloration was with a pale ring. Lateral median areas of enamel orientation were large. Eyes were distinctly convex. The internal spur length of Coxa I was short. Coxae II and III had a broad salient ridge-like spur. Coxa I external spur length was median. In addition, 16S rRNA and cox1 genes were used to confirm this species. *Amblyomma lepidum* was not detected in livestock tick samples from Abu Dhabi and Sharjah. DNA fragments were identified based on DNA sequence similarity with the records of cox1 gene from the GenBank (Supplementary Table S4). A representative sequence of *A. lepidum* was deposited in the GenBank (OK001821) (Table 2). This sequence was 99.84% identical to the *A. lepidum* from sheep in Israel (KP987775.1), and 99.38% similar to *A. lepidum* from Kenya (KT307492.1), with sequence coverage of 93% and 94%, respectively (Supplementary Table S4).

**Figure 5:**
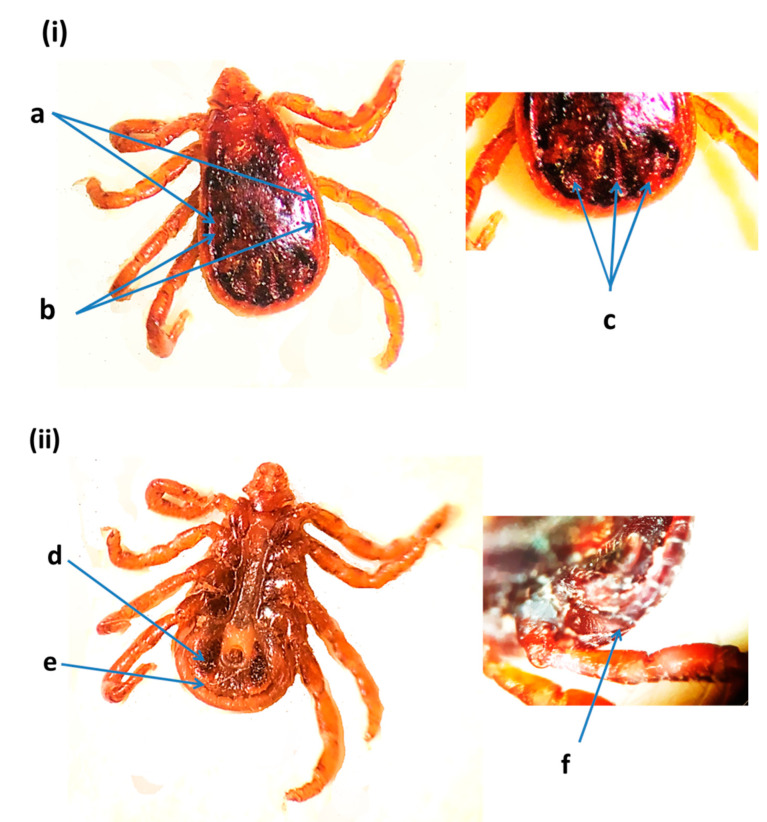
*Rhipicephalus sanguineus* male from the United Arab Emirates: (**i**) dorsal view: (a) marginal lines, (b) marginal lines anteriorly almost to eye level, (c) posterior grooves (comma shaped); (**ii**) ventral view: (d) adanal plates, (e) subadanal plates absent, (f) spiracle plate [11,29].

*Rhipicephalus sanguineus* is a brown colored tick and was present only on cows from Dubai. Diagnostic features are given in Figure 5. Marginal lines were heavily punctate. Furthermore, marginal lines were long, deep, and reaching anteriorly almost to eye level. There were three posterior grooves that were comma shaped, with one posteromedian groove and two posterolateral grooves. The adanal plates were curved but not sickle shaped. Subadanal plates were absent. The spiracle plate had a narrow tail. *Rhipicephalus sanguineus* was confirmed based on sequence similarities with GenBank records (Supplementary Table S5). A representative sequence of *R. sanguineus* from cows was submitted in the GenBank (MZ976769) (Table 2). This sequence showed 99.03% similarity to the sequences of *R. sanguineus* taken from dogs in India (MG066692.1), 98.56% from dogs in Taiwan (AY883868.1), and 98.33% from dogs in Cuba (KP830114.1), with sequence coverage of 96%, 98%, and 98%, respectively (Supplementary Table S5).

### 3.2. Tick Prevalence

**Figure 6:**
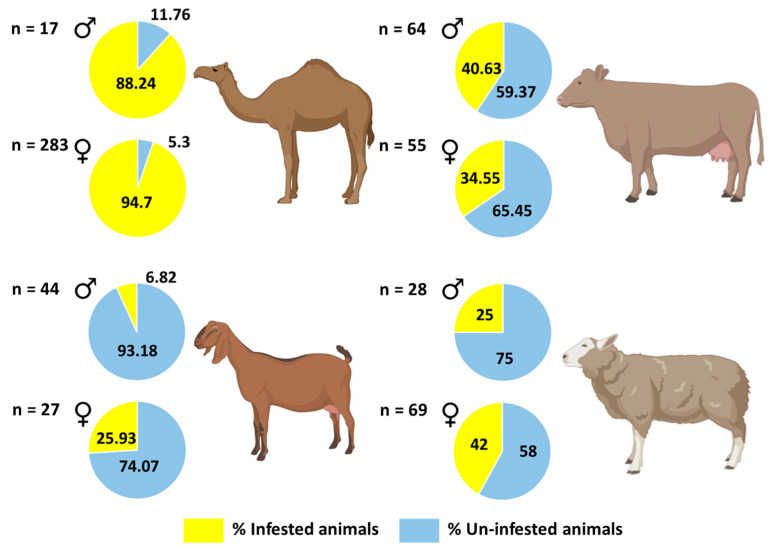
Prevalence of ticks per host (camel, cow, goat, and sheep) in relation to the sex of hosts, in 2019, 2020 and 2021, in the United Arab Emirates. The figure was created with BioRender (https://biorender.com/) (accessed on 4 August 2021).

The prevalence of ticks in camels (94%) was very high as compared to cows, sheep, and goats (Table 3) (Supplementary Table S6) (Fisher’s Exact test, *p* < 0.001 for all pairwise comparisons). The prevalence of ticks in cows (38%) was also high as compared to goats (14%) (Fisher’s Exact test, *p* < 0.001); however, tick prevalence did not differ significantly between cows (38%) and sheep (37%) (Fisher’s Exact test, *p* = 1.00). In addition, the prevalence of ticks in sheep (37.1%) was higher than that of goats (14%) (Fisher’s Exact test, *p* < 0.001). Prevalence of ticks did not differ significantly between male and female hosts (Fisher’s Exact test, *p* > 0.05 for all pairwise comparisons) except in goats, where prevalence was higher in females (25.9%) than males (6.8%) (Fisher’s Exact test, *p* = 0.036) (Figure 6; Table 3) (Supplementary Table S7). We did not find any ticks on Australian cows.

Mean intensity of ticks on camels was significantly higher than on sheep (Bootstrap 2-sample *t*-test, *p* < 0.001). However, mean intensity of ticks did not differ significantly between camels and cows, and between camels and goats (Bootstrap 2-sample *t*-test, *p* > 0.05 for all pairwise comparisons). Further, mean intensity of ticks on cows was significantly higher than on sheep (Bootstrap 2-sample *t*-test, *p* < 0.005 for pairwise comparison). There was no significant difference between mean intensities in pairwise comparisons between cows and goats, nor between sheep and goats (Bootstrap 2-sample *t*-test, *p* > 0.05).

Mean tick abundance on camels was significantly higher than on cows, sheep, and goats (Bootstrap 2-sample *t*-test, *p* < 0.001 for all pairwise comparisons). However, there was no difference in mean abundance of ticks on goats and sheep, and cows and goats (Bootstrap 2-sample *t*-test, *p* > 0.05 for all pairwise comparisons). Mean tick abundance on cows was significantly higher than that of sheep (Bootstrap 2-sample *t*-test, *p* < 0.01). *Hyalomma dromedarii* ticks were collected in large numbers only from camels in all three emirates with 94.3% prevalence (Table 4). *Hyalomma anatolicum* was found in all emirates on all hosts. The prevalence of *H. anatolicum* on cows (32.8%) was high compared to camels, goats, and sheep (Fisher’s Exact test, *p* < 0.01 for all pairwise comparisons). However, prevalence of *H. anatolicum* did not differ significantly between sheep and goats, nor between camels and goats (Fisher’s Exact test, *p* > 0.05 for all pairwise comparisons). Mean intensity and mean abundance of *H. anatolicum* in all hosts did not differ significantly (Bootstrap 2-sample *t*-test, *p* > 0.05 for all pairwise comparisons), except camels and cows (Bootstrap 2-sample *t*-test, *p* < 0.01). *Amblyomma lepidum* was recorded in cows from Dubai and *R. sanguineus* was recorded in cows from Sharjah, in very low numbers with 0.8% prevalence. The engorged nymphs and engorged female ticks, which were difficult to identify at the species level, were categorized as “others” (Supplementary Table S8).

## 4. Discussion

The main purpose of this study was to identify ticks associated with livestock and assess their prevalence. Morphological and molecular analyses of the ticks collected in the present study confirmed the occurrence of *H. dromedarii*, *H. anatolicum, A. lepidum* and *R. sanguineus*. In addition, the current work provides the first molecular identification of *H. anatolicum, A. lepidum*, and *R. sanguineus* along with morphological characterization in the UAE. *Rhipicephalus sanguineus* has often been reported from cattle in Iraq and Pakistan [36,37,38] and is presumably associated with dogs near farms or animal markets. Although the four tick species reported in this study can be morphologically distinguished relatively easily, some notable similarities might cause taxonomic confusion, underscoring the need for molecular identification. Some of the distinctive features of *R. sanguineus* collected in this study included the presence of three comma-shaped, posterior grooves and adanal plates that were curved. In addition, the subadanal plates were absent [11,29] as compared to other *Rhipicephalus* species. *Hyalomma dromedarii* was larger than *H. anatolicum* and the sub-anal plates were aligned outside the adanal plates in male ticks [11]. *Hyalomma anatolicum* were reddish brown in color and the sub-anal shields were situated on the axis of the adanals [11,31]. *Amblyomma lepidum*, identified in the current work, was a large and ornamented tick with pink to orange enamels and long mouth parts. The posteromedian strip was narrow, which separates it from *Amblyomma gemma* [11,30], in which the posteromedian stripe is broad [11]. Although diagnostic morphological characters can be used to identify ticks at the species level, the quality and age of the specimen could pose challenges in identification. Thus, molecular methods could improve or confirm morphological identification, especially for species that are difficult to identify. There having been previous records made of *H. dromedarii*, *H. anatolicum, A. lepidum,* and *R. sanguineus* infestations in neighboring countries, our findings shed light on the need for conducting joint projects on the tick species that are common in countries sharing borders. In saying that, however, further studies are apparently needed to better evaluate the possible occurrence of more tick species in the UAE.

Camels were heavily infested (94.3%) with *H. dromedarii* compared to all animals and these results concurred with our previous studies in the UAE [12,39]. *Hyalomma dromedarii* is known to harbor a variety of microbes with some tick-borne bacterial [25,40,41,42] and protozoan pathogens [25] in the MENA region, including the UAE. There was no difference in tick prevalence between male and female hosts with the exception of goats, where more females were infested compared to males. However, previously males (cattle) were found to be more infested (63.4%) than females (60.9%) in Nigeria [43]. Some studies recorded that the female cattle infestation rate was slightly higher (46.5%) than males (45%) [44]. In sheep, females were reported heavily infested (100%) compared to male sheep in Cameroon [45]. Selection of male and female hosts could be related to host behavior or odors; this observation requires further study to determine why, in some hosts, ticks show a preference for a particular sex.

We found *H. anatolicum* on all hosts in the sampling areas of the UAE, suggesting that the same tick species in the three emirates was probably associated with unrestricted livestock trade and the movement of animals between emirates [46]. Tick prevalence, mean intensity, and mean abundance varied among the four animal hosts (camel, cows, sheep, and goats) and this is most likely due to host preference in this species. Furthermore, *H. anatolicum* was the most prevalent tick in cows (32.8%) compared to other tick species. Our results are comparable to other studies that reported a high prevalence of *H. anatolicum* in cattle (63.1%) [47] and in livestock (38.83%) [48] as compared to other tick species. In addition, a very high prevalence of *H. anatolicum* in cattle and sheep has been reported from Iraq [36]. *Hyalomma anatolicum* was also found to be the most prevalent tick species in sheep (14.4%), followed by goats (9.9%). In contrast, in Cameroon [45] *Boophilus geygei* was reported as the most dominant species in goats. Such differences in the results may be due to different geographic areas and differences in climate in various regions. In the current study, we found a high prevalence of *H. anatolicum* on all hosts except camels. This could be attributed to farming conditions and resistance of this tick species to acaricides. This tick is the competent vector of CCHF [49] and poses a serious threat to livestock, as well as humans, who may be exposed to tick bites in the livestock industry. In the UAE, this species was found to be a carrier of CCHF [7], *T. annulata* and *Theileria ovis* [50].

*Rhipicephalus sanguineus* or the kennel tick was identified from cattle tick samples in Sharjah with very low prevalence (0.8%) and the results of the present study are comparable to records from Iraq where *R. sanguineus* prevalence was recorded as 0.09% in cattle and sheep during an investigation of monthly tick distribution [36]. In addition, *R. sanguineus* was recorded with a low prevalence (7.52%) on cattle [38] and on bovine species (13%) [37] from Pakistan. The higher prevalence of this tick on livestock could be associated with the generally higher abundance of stray dogs in those regions [37,38]. In the UAE, dogs are not present as feral or stray populations, and possibly this can prevent the buildup of the ticks in such populations.

In this study, *A. lepidum* was found only on cattle with low prevalence (0.8%) from Dubai. This is a common tick species on livestock in Sudan, Ethiopia, Somalia, Uganda, Kenya, and Tanzania [11]. It has a potential to cause bovine theileriosis and heart-water in livestock by transmitting bacterial and protozoan pathogens [11,51]. Though it was found to have the lowest prevalence, it is a very important species that could pose a risk to livestock if allowed to build up in numbers.

Although the current work reported the presence and distribution of four tick species, a well-documented distribution of ticks in the entire UAE is still needed. In fact, the true prevalence of ticks among livestock in many regions of the UAE is unknown. The presence of the aforementioned four tick species is of medical relevance because some of them are known vectors of diseases, such as CCHF and spotted fever group (SFG) *Rickettsia*. Pathogens carried by ticks can infect both livestock and people, and thus monitoring ticks and the pathogens they carry is very important in implementing control measures to combat tick-borne disease outbreaks. This study provides insight into the occurrence of multiple ticks and consequently contributes to the broad control efforts of ticks and their associated diseases in the UAE. The precise distribution of ticks, particularly *R. sanguineus* and *A. lepidum*, in the UAE requires further study.

## 5. Conclusions

The present study provides a DNA-based and morphological characterization as well as the prevalence and geographic distributions of four hard ticks (*H. dromedarii*, *H. anatolicum*, *R. sanguineus*, and *A. lepidum*) collected from livestock in three emirates in the UAE. The occurrence of different tick species reveals the possible diversity of the hard ticks present in the UAE. In addition, the presence of a tick species could indicate the potential presence of associated tick-borne pathogens when environmental and host conditions conducive for infection are present. Further surveillance is needed to collect and identify tick species in the other regions of the UAE. In particular, studies should focus on the poorly investigated tick species that are known to serve as reservoirs of important tick-borne pathogens.

## Figures and Tables

**Table 1:** Primers and thermo-cycle conditions used to amplify gene fragments.

Target Gene	Primer	Sequence (5′–3′)	Cycle Conditions	Amplicon Size (bp)	Reference
16S rRNA	16S + 1 16S − 1	CTGCTCAATGATTTTTTAAATTGCTGTGG CCGGTCTGAACTCAGATCAAGT	94 °C 5 min 32 cycles: 94 °C 1 min 52.9 °C 1 min 72 °C 1 min 72 °C 15 min	460	[33]
cox1	LCO1490 HCO2198	GGTCAACAAATCATAAAGATATTGG TAAACTTCAGGGTGACCAAAAAATCA	95 °C 5 min 30 cycles: 94 °C 1 min 54 °C 1 min 72 °C 1 min 30 s 72 °C 10 min	710	[34]

**Table 2:** Identity of tick species and percentage similarity value with the reference sequences from the GenBank.

Sample Accession	Host	Location	GenBank Reference		Identity%	Species
			16S	cox1		
MZ976772	Camel	Abu Dhabi	L34306.1	-	99.27	*H. dromedarii*
OK017169	Cow	Dubai	-	MT800311.1	99.70	*H. anatolicum*
MZ976771	Cow	Dubai	MK829042.1	-	99.28	*H. anatolicum*
MZ976770	Sheep	Dubai	MK829042.1	-	99.52	*H. anatolicum*
MZ976780	Goat	Dubai	KC203338.1	-	99.51	*H. anatolicum*
MZ976769	Cow	Sharjah	MG066692.1	-	99.03	*R. sanguineus*
OK001821	Cow	Dubai	-	KP987775.1	99.84	*A. lepidum*

**Table 3:** Tick prevalence, mean intensity, mean abundance on camels, cows, sheep, and goats in the sampling areas, UAE.

Hosts	Examined Animals	Infested with Ticks	Prevalence (95% Confidence Level)	Mean Intensity (95% Confidence Level)	Mean Abundance (95% Confidence Level)
Camels	300	283	0.94 (0.91–0.97)	17 (15.23–19.52)	16 (14.39–18.63)
Cows	119	45	0.38 (0.29–0.47)	14.47 (11.18–18.87)	5.47 (3.99–7.87)
Sheep	97	36	0.37 (0.28–0.48)	7.69 (5.69–10.67)	2.85 (1.96–4.36)
Goats	71	10	0.14 (0.07–0.24)	21.9 (10.50–55)	3.08 (1.14–9.08)

**Table 4:** Number (n) of tick species collected from camels, cows, sheep, and goats in the sampling areas, UAE.

Hosts	Camels			Cows			Sheep			Goats		
	P (%)	MI (M ± SE)	MA (M ± SE)	P (%)	MI (M ± SE)	MA (M ± SE)	P (%)	MI (M ± SE)	MA (M ± SE)	P (%)	MI (M ± SE)	MA (M ± SE)
*H. dromedarii*	94.3	16.52 ± 1.05	15.58 ± 1.01	0	0	0	0	0	0	0	0	0
*H. anatolicum*	3.7	1.18 ± 0.12	0.04 ± 0.01	32.8	8.51 ± 1.34	2.79 ± 0.57	14.4	10.36 ± 3.25	1.5 ± 0.59	9.9	15.14 ± 10.38	1.49 ± 1.10
*R. sanguineus*	0	0	0	0.8	5	0.04 ± 0.04	0	0	0	0	0	0
*A. lepidum*	0	0	0	0.8	2	0.02 ± 0.02	0	0	0	0	0	0
Others	8	4.83 ± 0.56	0.39 ± 0.09	36.1	7.26 ± 0.96	2.62 ± 0.47	34	4 ± 1.01	1.4 ± 0.39	14.1	11.7 ± 3.53	1.65 ± 0.68

P = prevalence, MI = Mean intensity, MA = Mean Abundance.

## Data Availability

Data is contained within the article or Supplementary Material.

## Supplementary Materials

The following are available online at https://www.mdpi.com/article/10.3390/insects12111016/s1: Table S1: Tick collection sites and distribution of tick species on livestock in the study area, in 2019, 2020 and 2021, in the United Arab Emirates. Table S2: Molecular identification of *Hyalomma dromedarii* from camels in Abu Dhabi, United Arab Emirates based on DNA similarity between 16S rRNA gene and GenBank species using NCBI BLAST. Table S3: Molecular identification of *Hyalomma anatolicum* from cows in Dubai, United Arab Emirates based on DNA similarity between cox1 gene and GenBank species using NCBI BLAST. Table S4: Molecular identification of *Amblyomma lepidum* from cows in Dubai, United Arab Emirates, based on DNA similarity between cox1 gene and GenBank species using NCBI BLAST. Table S5: Molecular identification of *Rhipicephalus sanguineus* from cows in Sharjah, United Arab Emirates based on DNA similarity between 16S rRNA gene and GenBank species using NCBI BLAST. Table S6: Prevalence of ticks in camels, cows, sheep and goats in the United Arab Emirates^a^. Table S7: Prevalence of ticks in camels, cows, sheep and goats in relation to sex of animals in the United Arab Emirates^b^. Table S8: Number (N) of tick species collected from camels, cows, sheep and goats in the United Arab Emirates.
