# Supplementary material for: Prevalence, Distribution, and Molecular Record of Four Hard Ticks from Livestock in the United Arab Emirates
Source: Insects. 2021 Nov 11;12(11):1016. doi: 10.3390/insects12111016 (PMC8617910; doi:10.3390/insects12111016)
Supplement: Supplementary file 1 [file insects-12-01016-s001.zip › insects-1388957-supplementary.pdf]

Supplementary Table S1. Tick collection sites and distribution of tick species on livestock in the study area, in 2019, 2020 and 2021, in the United Arab Emirates.

**Sr. No.**	**Farm Locations**	**Camels (n=300)**			**Cows (n=119)**			**Sheep (n=97)**			**Goats (n=71)**			***Hyalomma dromedarii***			****Hyalomma anatolicum***			*****Amblyomma lepidum***			******Rhipicephalus sanguineus***		
Abu Dhabi		2019	2020	2021	2019	2020	2021	2019	2020	2021	2019	2020	2021	2019	2020	2021	2019	2020	2021	2019	2020	2021	2019	2020	2021
1.	Al-Yahar	5 (6)	-	-	-	-	-	15 (30)	-	-	-	-	-	58	-	-	104	-	-	-	-	-	-	-	-
2.	Remah	5 (5)	-	-	-	-	-	-	-	-	-	-	-	33	-	-	-	-	-	-	-	-	-	-	-
3.	Al-Hayer	5 (5)	-	-		-	-		-	-	-	-	-	122	-	-	-	-	-	-	-	-	-	-	-
4.	Nahel	18 (21)	-	-	-	-	-	-	-	-	-	-	-	740	-	-	-	-	-	-	-	-	-	-	-
5.	DR	29 (35)	-	-	0 (14)	-	-	-	-	-		-	-	347	-	-	-	-	-	-	-	-	-	-	-
6.	BBS	6 (6)	-	-	-	-	-	-	-	-	-	-	-	73	-	-	-	-	-	-	-	-	-	-	-
7.	Swehan	16 (16)	-	-	-	-	-	-	-	-	0 (15)	-	-	217	-	-	-	-	-	-	-	-	-	-	-
8.	Al-Saad	16 (16)	10 (10)	-		-	-		-	-	-	-	-	200	98	-	7	-	-	-	-	-	-	-	-
9.	Beda Fares	17(17)	-	-	-	-	-		-	-	-	-	-	102	-	-	-	-	-	-	-	-	-	-	-
10.	LSM,AlAin	9 (11)	-	-	-	-	-	11 (30)	-	-	0 (15)	-	-	114	-	-	90	-	-	-	-	-	-	-	-
11.	TR	20 (20)	-	-	-	-	-	-	-	-	-	-	-	148	-	-	-	-	-	-	-	-	-	-	-
12.	Seh Saba	3 (3)	-	-	-	-	-	-	-	-	-	-	-	9	-	-	-	-	-	-	-	-	-	-	-
13.	Malaket	12 (12)	-	-	-	-	-	-	-	-	-	-	-	408	-	-	-	-	-	-	-	-	-	-	
14.	Omghafa	15 (15)	8(8)	-	-	-	-	-	-	-	-	-	-	364	61	-	-	-	-	-	-	-	-	-	-
15.	Al-Dahra	19 (19)	9(9)	-		-	-	-	-	-	-	-	-	497	123	-	6	-	-	-	-	-	-	-	-
16.	Bukriya	15 (15)	-	-	-	-	-	-	-	-	-	-	-	245	-	-	-	-	-	-	-	-	-	-	
17.	Al-Arad	10 (10)	-	-	-	-	-	-	-	-	-	-	-	144	-	-	-	-	-	-	-	-	-	-	-
18.	Al-Wagan	18 (18)	-	-	-	-	-	-	-	-	-	-	-	312	-	-	-	-	-	-	-	-	-	-	-
S. total		265 (277)			0 (14)			26 (60)			0 (30)			4415			207			0			0		
Dubai																									
19.	DCH	17 (20)			-	-	-	-	-	-	-	-	-	248	-	-	-	-	-	-	-	-	-	-	-
20.	LSMQusais	-			43(93)			9 (30)			9 (35)						230	-	64	-	-	2			
S. total		17 (20)			43 (93)			9 (30)			9 (35)			248			294			2			0		
Sharjah																									
21.	Kalba	1 (3)			2 (12)			1 (7)			1 (6)			11			76	-	-	-	-	-	5	-	-
S. total		1 (3)			2 (12)			1 (7)			1 (6)			11			76			-			5		
Total		283(300)			45 (119)			36 (97)			10 (71)			4674			577			2			5		

DR=Dubai road, BBS=Bede Bent Saud, TR=Truck road, LSM=Livestock market, DCH=Dubai camel hospital

In parentheses, total number of examined animals are given

**Hyalomma dromedarii*=all ticks collected from camels, ***Hyalomma anatolicum*= ticks collected from cows, sheep and goats

****Amblyomma lepidum*= ticks collected from cows, *****Rhipicephalus sanguineus*= ticks collected from cows

Supplementary Table S2. Molecular identification of *Hyalomma dromedarii* from camels in Abu Dhabi, United Arab Emirates based on DNA similarity between 16S rRNA gene and GenBank species using NCBI BLAST.

Best match species	Accession number	Sequence Identity %	Sequence coverage %	*E*-value	Host	Country
Mitochondrion *Hyalomma dromedarii* 16S ribosomal RNA (16S rRNA) gene	L34306.1	99.27	98	0.0		
Hyalomma dromedarii voucher HyT85 large subunit ribosomal RNA gene, partial sequence; mitochondrial	MN960589.1	99.50	95	0.0	Camel	Tunisia
Hyalomma dromedarii 16S ribosomal RNA gene, partial sequence; mitochondrial	MG757400.1	99.50	95	0.0	Camel	Egypt
Hyalomma dromedarii voucher HyT1 large subunit ribosomal RNA gene, partial sequence; mitochondrial	MN960579.1	99.49	94	0.0	Camel	Tunisia
Hyalomma dromedarii 16S ribosomal RNA gene, partial sequence; mitochondrial	MG972372.1	98.53	96	0.0	Camel	Saudi Arabia
Hyalomma dromedarii isolate Gebal_92 16S ribosomal RNA gene, partial sequence; mitochondrial	KY512798.1	99.49	93	0.0	*Dipodillus dasyurus*	Egypt
Hyalomma dromedarii isolate Gharaba _13/11 16S ribosomal RNA gene, partial sequence; mitochondrial	KY512796.1	99.49	93	0.0	Camel	Egypt
Hyalomma dromedarii isolate MATRUH-EGY 16S ribosomal RNA gene, partial sequence; mitochondrial	MF946465.1	98.99	94	0.0	Camel	Egypt
Hyalomma dromedarii isolate Marsa-Matruh 16S ribosomal RNA gene, partial sequence; mitochondrial	KY945490.1	98.99	94	0.0	Camel	Egypt
Hyalomma dromedarii isolate Arbaein_15/3 16S ribosomal RNA gene, partial sequence; mitochondrial	KY512797.1	99.23	93	0.0		Egypt
Hyalomma dromedarii strain ST6HD2 large subunit ribosomal RNA gene, partial sequence; mitochondrial	MH569481.1	99.74	91	0.0	Camel	Saudi Arabia
Hyalomma dromedarii voucher HyT48 large subunit ribosomal RNA gene, partial sequence; mitochondrial	MN960587.1	99.23	93	0.0	Camel	Tunisia
Hyalomma dromedarii voucher HyT7 large subunit ribosomal RNA gene, partial sequence; mitochondrial	MN960580.1	99.48	91	0.0	Camel	Tunisia
Hyalomma dromedarii strain ST3HD35 large subunit ribosomal RNA gene, partial sequence; mitochondrial	MH569480.1	99.48	91	0.0	Camel	Saudi Arabia
Hyalomma dromedarii strain ST2HD44 large subunit ribosomal RNA gene, partial sequence; mitochondrial	MH569479.1	99.48	91	0.0	Camel	Saudi Arabia
Hyalomma dromedarii strain ST5HD50 large subunit ribosomal RNA gene, partial sequence; mitochondrial	MH569478.1	99.48	91	0.0	Camel	Saudi Arabia

Hyalomma dromedarii strain ST1HD46 large subunit ribosomal RNA gene, partial sequence; mitochondrial	MH569477.1	99.48	91	0.0	Camel	Saudi Arabia
Hyalomma dromedarii strain ST4HD14 large subunit ribosomal RNA gene, partial sequence; mitochondrial	MH569476.1	99.48	91	0.0	Camel	Saudi Arabia
Hyalomma dromedarii isolate Hdrom4 16S ribosomal RNA gene, partial sequence; mitochondrial	KU130425.1	99.74	90	0.0		Senegal
Hyalomma dromedarii isolate Hdrom2 16S ribosomal RNA gene, partial sequence; mitochondrial	KU130423.1	99.74	90	0.0		Pakistan
Hyalomma dromedarii isolate Hdrom3 16S ribosomal RNA gene, partial sequence; mitochondrial	KU130424.1	99.47	90	0.0		Saudi Arabia
Hyalomma dromedarii isolate KNC106 large subunit ribosomal RNA gene, partial sequence; mitochondrial	MN394434.1	99.21	90	0.0	Camel	Nigeria
Hyalomma dromedarii voucher MT1 large subunit ribosomal RNA gene, partial sequence; mitochondrial	MT895170.1	99.73	89	0.0	Camel	Kenya
Hyalomma dromedarii voucher MT120 large subunit ribosomal RNA gene, partial sequence; mitochondrial	MT895169.1	99.73	89	0.0	Camel	Kenya
Hyalomma dromedarii isolate Hdrom1 16S ribosomal RNA gene, partial sequence; mitochondrial	KU130422.1	99.21	90	0.0		Iraq
Hyalomma dromedarii isolate KNC19 large subunit ribosomal RNA gene, partial sequence; mitochondrial	MN394429.1	100.00	88	0.0	Camel	Nigeria
Hyalomma dromedarii voucher HyT41 large subunit ribosomal RNA gene, partial sequence; mitochondrial	MN960585.1	97.95	93	0.0	Camel	Tunisia
Hyalomma dromedarii isolate KNC21 large subunit ribosomal RNA gene, partial sequence; mitochondrial	MN394430.1	100.00	86	0.0	Camel	Nigeria
Hyalomma dromedarii isolate KNC47 large subunit ribosomal RNA gene, partial sequence; mitochondrial	MN394431.1	99.45	86	0.0	Camel	Nigeria
Hyalomma somalicum isolate Hsoma1 16S ribosomal RNA gene, partial sequence; mitochondrial	KU130472.1	98.15	90	0.0		Somalia
Hyalomma dromedarii isolate KNC13 large subunit ribosomal RNA gene, partial sequence; mitochondrial	MN394427.1	98.90	87	0.0	Camel	Nigeria
Hyalomma dromedarii isolate KNC101 large subunit ribosomal RNA gene, partial sequence; mitochondrial	MN394433.1	99.72	85		Camel	Nigeria
Hyalomma dromedarii isolate Hydr1 16S ribosomal RNA gene, partial sequence; mitochondrial	KT391055.1	97.17	91			Israel

Supplementary Table S3. Molecular identification of *Hyalomma anatolicum* from cows in Dubai, United Arab Emirates based on DNA similarity between cox1 gene and GenBank species using NCBI BLAST.

Best match species	Accession number	Sequence Identity %	Sequence coverage %	*E*-value	Host	Country
Hyalomma anatolicum anatolicum isolate COX1	MT800311.1	99.70	96	0.0	Goat	Pakistan
Hyalomma anatolicum voucher AC9 cytochrome oxidase subunit 1 (COI) gene	MH459380.1	99.39	97	0.0	Cattle	China
Hyalomma anatolicum anatolicum isolate 3old cytochrome oxidase subunit I (Cox1) gene	KP792577.1	99.85	95	0.0	Buffalo	India
Hyalomma anatolicum isolate GY44-2 cytochrome oxidase subunit I (COI) gene	MN853167.1	99.24	97	0.0	Cattle	China
Hyalomma anatolicum isolate GY43-1 cytochrome oxidase subunit I (COX1) gene	MN841463.1	99.24	97	0.0	Cattle	China
Hyalomma anatolicum voucher AC5 cytochrome oxidase subunit 1 (COI) gene	MH459377.1	99.24	97	0.0	Cattle	China
Hyalomma anatolicum anatolicum cytochrome oxidase subunit I (COI) gene	KJ912622.2	99.85	95	0.0	Cattle	India
Hyalomma anatolicum anatolicum isolate Gansu cytochrome oxidase subunit I (COI) gene	JQ737067.1	99.24	97	0.0	Cattle	China
Hyalomma anatolicum isolate PAK5 cytochrome c oxidase subunit 1 (cox1) gene	MK462197.1	99.69	95	0.0	Cattle	Pakistan
Hyalomma anatolicum isolate XJ-TLF-Han-2019001 cytochrome c oxidase subunit I (COX1) gene	MW221948.1	99.24	96	0.0	Cattle	China
Hyalomma anatolicum isolate XJ074 cytochrome c oxidase subunit I gene	KF583577.1	99.39	96	0.0	Cattle	China
Hyalomma anatolicum voucher ACc cytochrome oxidase subunit 1 (COI) gene	MH459383.1	99.09	97	0.0	lab rearing	China
Hyalomma anatolicum isolate PACA-83 cytochrome c oxidase subunit 1 (cox1) gene	MK462202.1	99.54	95	0.0	Cattle	Pakistan
Hyalomma anatolicum isolate PACA-116 cytochrome c oxidase subunit 1 (cox1) gene	MK462200.1	99.54	95	0.0	Cattle	Pakistan
Hyalomma anatolicum isolate PACA-88 cytochrome c oxidase subunit 1 (cox1) gene	MK462199.1	99.54	95	0.0	Cattle	Pakistan
Hyalomma anatolicum isolate PAK6 cytochrome c oxidase subunit 1 (cox1) gene,	MK462198.1	99.54	95	0.0	Cattle	Pakistan
Hyalomma anatolicum voucher TK0G9 cytochrome oxidase subunit 1 (CO1) gene	MH648685.1	99.69	94	0.0	Cattle	Bangladesh

Supplementary Table S4. Molecular identification of *Amblyomma lepidum* from cows in Dubai, United Arab Emirates, based on DNA similarity between cox1 gene and GenBank species using NCBI BLAST.

Best match species	Accession number	Sequence Identity %	Sequence coverage %	*E*-value	Host	Country
Amblyomma lepidum cytochrome c oxidase subunit 1 (COX1) gene, partial cds; mitochondrial	KP987775.1	99.84	93	0.0	*Ovis aries*	Israel
Amblyomma lepidum voucher 19634-AlepB10 cytochrome oxidase subunit 1 (COI) gene, partial cds; mitochondrial	KT307492.1	99.38	94	0.0		Kenya
Amblyomma cohaerens isolate AET3 cytochrome c oxidase subunit 1 (cox1) gene, partial cds; mitochondrial	MN150170.1	88.47	91	0.0	*Bos taurus*	Ethiopia
Amblyomma cohaerens isolate AET4 cytochrome c oxidase subunit 1 (cox1) gene, partial cds; mitochondrial	MN150171.1	88.31	91	0.0	*Bos taurus*	Ethiopia
Amblyomma testudinarium voucher AMMS-AF-2-1 cytochrome c oxidase subunit I-like (COI) gene, partial sequence; mitochondrial	HM193893.1	86.39	97	0.0		China
Amblyomma pattoni voucher AMMS-AP-2 cytochrome c oxidase subunit I-like (COI) gene, partial sequence; mitochondrial	HM193876.1	86.43	96	0.0		China
Amblyomma hebraeum isolate 190 cytochrome c oxidase subunit I (COX1) gene, partial cds; mitochondrial	MT549815.1	86.65	93	0.0		China
Amblyomma hebraeum isolate 200 cytochrome c oxidase subunit I (COX1) gene, partial cds; mitochondrial	MT549816.1	86.34	93	0.0		China
Amblyomma testudinarium isolate TWKL-Amt1 cytochrome c oxidase subunit I (COI) gene, partial cds; mitochondrial	KX712282.1	86.15	94	0.0		Taiwan
Amblyomma testudinarium isolate TWKL-Amt3 cytochrome c oxidase subunit I (COI) gene, partial cds; mitochondrial	KX712284.1	86.00	94	0.0		Taiwan
Amblyomma testudinarium isolate TWKL-Amt2 cytochrome c oxidase subunit I (COI) gene, partial cds; mitochondrial	KX712283.1	86.00	94	0.0		Taiwan
Amblyomma variegatum isolate 1 cytochrome c oxidase subunit I (COX1) gene, partial cds; mitochondrial	MT549807.1	85.49	93	0.0		China
Amblyomma variegatum isolate 11 cytochrome c oxidase subunit I (COX1) gene, partial cds; mitochondrial	MT549808.1	85.34	93	0.0		China
Amblyomma pattoni voucher AMMS-AP-5 cytochrome c oxidase subunit I-like (COI) gene, partial sequence; mitochondrial	HM193875.1	85.69	91	0.0		China
Amblyomma scalpturatum isolate scalpturatumN2921 cytochrome oxidase subunit 1 (COX1) gene, partial cds; mitochondrial	MH513238.1	84.98	93	0.0		French Guiana

Amblyomma scalpturatum isolate scalpturatumN4222 cytochrome oxidase subunit 1 (COX1) gene, partial cds; mitochondrial	MH513239.1	84.83	93	0.0		French Guiana
Amblyomma variegatum isolate ANI1 cytochrome c oxidase subunit 1 (cox1) gene, partial cds; mitochondrial	MN150168.1	85.17	91	0.0	*Canis familiaris*	Nigeria

Supplementary Table S5. Molecular identification of *Rhipicephalus sanguineus* from cows in Sharjah, United Arab Emirates based on DNA similarity between 16S rRNA gene and GenBank species using NCBI BLAST.

Best match species	Accession number	Sequence Identity %	Sequence coverage %	*E*-value	Host	Country
Rhipicephalus sanguineus clone TVM small subunit ribosomal RNA gene, partial sequence; mitochondrial	MG066692.1	99.03	96	0.0	Dog	India
Rhipicephalus sanguineus strain 7 16S ribosomal RNA gene, partial sequence	AY883868.1	98.56	98	0.0	Dog	Taiwan
Rhipicephalus sanguineus isolate 3 16S ribosomal RNA gene, partial sequence; mitochondrial	MH765331.1	98.79	97	0.0	Goat	India
Rhipicephalus sanguineus isolate Bejucal 16S ribosomal RNA gene, partial sequence; mitochondrial	KP830114.1	98.33	98	0.0	Dog	Cuba
Rhipicephalus sanguineus 16S ribosomal RNA gene, partial sequence; mitochondrial	KC170744.1	98.33	98	0.0	Dog	Thailand
Rhipicephalus sanguineus isolate InDRE large subunit ribosomal RNA gene, partial sequence; mitochondrial	MT322611.1	98.33	98	0.0	Dog	Mexico
Rhipicephalus sanguineus strain 13 16S ribosomal RNA gene, partial sequence	AY883871.1	98.33	98	0.0	Dog	Taiwan
Rhipicephalus sanguineus strain 12 16S ribosomal RNA gene, partial sequence	AY883870.1	98.33	98	0.0	Dog	Taiwan
Rhipicephalus sanguineus strain 40 16S ribosomal RNA gene, partial sequence	AY883880.1	98.33	98	0.0	Dog	Taiwan
Rhipicephalus sanguineus strain 5 16S ribosomal RNA gene, partial sequence	AY883866.1	98.09	98	0.0	Dog	Taiwan
Rhipicephalus sanguineus isolate 2 large subunit ribosomal RNA gene, partial sequence; mitochondrial	MT026919.1	99.01	95	0.0	Mammals	Colombia
Rhipicephalus sanguineus strain 2 16S ribosomal RNA gene, partial sequence	AY883863.1	98.09	98	0.0	Dog	Taiwan

Rhipicephalus sanguineus isolate Colombia-Leticia 16S ribosomal RNA gene, partial sequence; mitochondrial	MF351600.1	98.09	98	0.0	Domestic animals	Colombia
Rhipicephalus sanguineus isolate Brazil-Minas Gerais 16S ribosomal RNA gene, partial sequence; mitochondrial	MF351603.1	98.31	97	0.0	Domestic animals	Colombia
Rhipicephalus sanguineus strain 32 16S ribosomal RNA gene, partial sequence	AY883878.1	97.86	98	0.0	Dog	Taiwan
Rhipicephalus sanguineus isolate Colombia-Saldana 16S ribosomal RNA gene, partial sequence; mitochondrial	MF351583.1	98.53	96	0.0	Domestic animals	Colombia
Rhipicephalus sanguineus isolate Colombia-Ibague16S ribosomal RNA gene, partial sequence; mitochondrial	MF351580.1	98.53	96	0.0	Domestic animals	Colombia
Rhipicephalus sanguineus haplotype A 16S ribosomal RNA gene, partial sequence; mitochondrial	KC476294.1	99.01	94	0.0	Dog	Nigeria
Rhipicephalus sanguineus isolate Colombia-Puerto Salgar 16S ribosomal RNA gene, partial sequence; mitochondrial	MF351595.1	98.07	97	0.0	Domestic animals	Colombia
Rhipicephalus sanguineus isolate Colombia-Cali(39-34V3C1) 16S ribosomal RNA gene, partial sequence; mitochondrial	MF351591.1	98.07	97	0.0	Domestic animals	Colombia
Rhipicephalus sanguineus isolate Colombia-Yopal 16S ribosomal RNA gene, partial sequence; mitochondrial	MF351588.1	98.07	97	0.0	Domestic animals	Colombia
Rhipicephalus sanguineus isolate IqRam 16S ribosomal RNA gene, partial sequence; mitochondrial	KT382453.1	99	94	0.0	Dog	Iraq
Rhipicephalus sanguineus isolate RSRC3 16S ribosomal RNA gene, partial sequence; mitochondrial	JX997392.1	98.52	95	0.0	Lab.colony	Brazil
Rhipicephalus sanguineus isolate RSJ1 16S ribosomal RNA gene, partial sequence; mitochondrial	JX997391.1	98.52	95	0.0	Lab.colony	Brazil
Rhipicephalus sanguineus 16S ribosomal RNA gene, partial sequence; mitochondrial	KU198404.1				Dog	Egypt
Rhipicephalus sanguineus isolate Orkun-RS314 small subunit ribosomal RNA gene, partial sequence; mitochondrial	KR870984.1				Dog	Turkey

Supplementary Table S6. Prevalence of ticks in camels, cows, sheep and goats in the United Arab Emirates[a].

Hosts	Type				Total
	Camels N (%)	Cows N (%)	Sheep N (%)	Goats N (%)	
Examined animals	300	119	97	71	587
Infested with ticks	283 (94.3)	45 (37.81)	36 (37.1)	10 (14)	374 (63.71)

[a] Number of infested animals/Number of examined animal X 100.

Supplementary Table S7. Prevalence of ticks in camels, cows, sheep and goats in relation to sex of animals in the United Arab Emirates[b].

Host	Type							
	Camels N (%)		Cows N (%)		Sheep N (%)		Goats N (%)	
	Male	Female	Male	Female	Male	Female	Male	Female
Examined animals	17	283	64	55	28	69	44	27
Infested with ticks	15 (88.24)	268 (94.7)	26 (40.63)	19 (34.55)	7 (25)	29 (42)	3 (6.82)	7 (25.93)

[b] Number of infested animals/Number of examined animal X 100.

Supplementary Table S8. Number (N) of tick species collected from camels, cows, sheep and goats in the United Arab Emirates.

Tick species	Hosts				Total
	Camels (N)	Cows (N)	Sheep (N)	Goats (N)	
Amblyomma lepidum	0	2	0	0	2
Hyalomma anatolicum	13	317	145	102	577
Hyalomma dromedarii	4674	0	0	0	4674
Rhipicephalus sanguineus	0	5	0	0	5
Others	116	327	132	117	692
Total	4803	651	277	219	5950
